# Application of potassium nitrate and salicylic acid improves grain yield and related traits by delaying leaf senescence in *Gpc-B1* carrying advanced wheat genotypes

**DOI:** 10.3389/fpls.2023.1107705

**Published:** 2023-07-17

**Authors:** Mohammad Jafar Tanin, Achla Sharma, Hari Ram, Satinder Singh, Puja Srivastava, G. S. Mavi, Dinesh Kumar Saini, Santosh Gudi, Pradeep Kumar, Prinka Goyal, V. S. Sohu

**Affiliations:** Department of Plant Breeding and Genetics, College of Agriculture, Punjab Agricultural University, Ludhiana, Punjab, India

**Keywords:** salicylic acid, potassium nitrate, *Gpc-B1* gene, isogenic lines, wheat

## Abstract

Grain protein content (GPC) is an important quality trait that effectively modulates end-use quality and nutritional characteristics of wheat flour-based food products. The *Gpc-B1* gene is responsible for the higher protein content in wheat grain. In addition to higher GPC, the *Gpc-B1* is also generally associated with reduced grain filling period which eventually causes the yield penalty in wheat. The main aim of the present study was to evaluate the effect of foliar application of potassium nitrate (PN) and salicylic acid (SA) on the physiological characteristics of a set of twelve genotypes, including nine isogenic wheat lines carrying the *Gpc-B1* gene and three elite wheat varieties with no *Gpc-B1* gene, grown at wheat experimental area of the Department of Plant Breeding and Genetics, PAU, Punjab, India. The PN application significantly increased the number of grains per spike (GPS) by 6.42 grains, number of days to maturity (DTM) by 1.03 days, 1000-grain weight (TGW) by 1.97 g and yield per plot (YPP) by 0.2 kg/plot. As a result of PN spray, the flag leaf chlorophyll content was significantly enhanced by 2.35 CCI at anthesis stage and by 1.96 CCI at 10 days after anthesis in all the tested genotypes. Furthermore, the PN application also significantly increased the flag leaf nitrogen content by an average of 0.52% at booting stage and by 0.35% at both anthesis and 10 days after anthesis in all the evaluated genotypes. In addition, the yellow peduncle colour at 30 days after anthesis was also increased by 19.08% while the straw nitrogen content was improved by 0.17% in all the genotypes. The preliminary experiment conducted using SA demonstrated a significant increase in DTM and other yield component traits. The DTM increased by an average of 2.31 days, GPS enhanced by approximately 3.17 grains, TGW improved by 1.13g, and YPP increased by 0.21 kg/plot. The foliar application of PN and SA had no significant effect on GPC itself. The findings of the present study suggests that applications of PN and SA can effectively mitigate the yield penalty associated with *Gpc-B1* gene by extending grain filling period in the wheat.

## Introduction

Wheat (*Triticum aestivum* L.) is the second most widely cultivated crop in the world that is consumed on a daily basis in many parts of the world. Additionally, during the 2019–2020 growing season, total global wheat production and productivity reached records of 764 million metric tonnes and 3.52 metric tonnes per hectare, respectively (https://worldpopulationreview.com/country-rankings/wheat-production-by-country). The protein content of harvested wheat grains is one of the most important quality traits as it significantly modulates the quality of the final food product(s). The world population is growing at an alarming rate and providing enough nutritionally enriched food for this rapidly expanding population will be a critical challenge for breeders. The recommended daily protein amount required for adults (more than 18 years old) is 0.8 g per kg or human weight (Wolfe et al., 2017). Acceptable protein-enriched food intake is a prerequisite factor for meeting human metabolic requirements and ensuring standard health criteria, especially in light of the current climate change crisis (Tanin et al., 2022b).

Grain protein content (GPC) is one of the highly valuable grain quality characteristics that determine the processing quality, nutritional capacity, and grain market properties. Breeders have made several attempts to improve the GPC in wheat using conventional breeding methods, but the desired results have not been achieved. This could be due to: (1) the substantial effect of the environment on GPC; (2) the negative relationship of GPC with grain yield; and (3) the complex quantitative genetic control of GPC and its low heritability. QTLs for GPC have been identified and mapped on nearly all chromosomes of wheat (Shewry, 2009; Saini et al., 2020; Saini et al., 2022b). *Gpc-B1* is the most critical QTL identified for GPC so far (Uauy et al., 2006; Saini et al., 2022b; Singh K. et al., 2022, http://www.wheatqtldb.net/). This QTL was discovered in a wild accession of tetraploid wheat, *Triticumturgidum* var. *dicoccoides* (Uauy et al., 2006). Later, the same accession (i.e., FA15-3) was used to generate a complete set of chromosome substitution lines with the background of modern durum wheat (Joppa and Cantrell, 1990). The *Gpc-B1* gene was then mapped on chromosome arm 6BS, which explained 66% of the phenotypic variation of the GPC and significantly improved GPC by an average of 14 g kg^-1^ in hexaploid wheat and by an average of 3.3 g kg^-1^ in tetraploid wheat under different environmental conditions (Uauy et al., 2006). The map-based cloning of *Gpc-B1* revealed that encodes a NAC transcription factor (*NAM-B1*).

The *NAM-B1*/*Gpc-B1* gene has been found to be associated with early senescence, improved nitrogen remobilization from flag leaf to the developing grains, and pleiotropic effects on protein accumulation in wheat grains, yield, and key yield-related traits, including, 1000-grain weight (TGW), number of spikes, number of spikelets per spike, and number of grains per spike (Uauy et al., 2006; Tabbita et al., 2017). The introgression of the functional allele of the *Gpc-B1* gene into the background of advanced wheat genotypes has resulted in the release of numerous cultivars in different countries (see Tabbita et al., 2017). However, GPC enhancement in genotypes with the *Gpc-B1* gene in their genetic background has been associated with a reduction in grain size and eventually the grain yield. In other words, the increased GPC could be attributed to more efficient nutrient transport from flag leaf to developing grains. The association of the *Gpc-B1* gene with accelerated senescence could explain both mechanisms. Even in the optimum grain filling environment, the TGW could be reduced due to a shorter period of grain filling, but earlier senescence may result in more effective nutrient transportation due to earlier initiation of the translocation process (Kade et al., 2005; Tanin et al., 2018).

The higher chlorophyll degradation in flag leaf, the lower spike water content, and the increased percentage of yellow peduncle have also been found to be associated with *Gpc-B1* (Uauy et al., 2006). Plants with delayed senescence are known as “stay-green”, and cosmetic types result from impeded chlorophyll catabolism (Thomas and Ougham, 2014; Kamal et al., 2019). In multiple crops, functional stay-green phenotypes have been found to be associated with increased or prolonged photosynthetic activities, conferring tolerance to drought, heat, and low nitrogen stresses (Thomas and Ougham, 2014). Significant positive correlation between green canopy and grain filling duration (Luche et al., 2016; Pinto et al., 2016) endorse the potential breeding versatility of stay-green traits, as a prolonged grain filling period that may boost grain size and final grain yield.

Potassium nitrate (PN, KNO_3_) is an inorganic salt that regulates different biochemical and physiological processes which influence plant growth and metabolism, including protein accumulation in grains, seed germination and emergence, stomatal regulation, phloem transport, cation-anion balance, photosynthesis, energy transfer, nutrient balance, and carbohydrate metabolism (Hasanuzzaman et al., 2018). The foliar application of PN showed the highest plant height, tiller number, spike length, and yield in an earlier study (Devi et al., 2017). In this study, uptake of nitrogen, phosphorus, and potassium was observed at the highest level with the PN spray at a rate of 3%. In another study, foliar spray of 0.5% PN at booting and anthesis stages increased the yield and yield component traits of wheat genotype HD2985 under late sown conditions (Singh and Singh, 2020). Similarly, salicylic acid (SA, C_7_H_6_O_3_) is a phenolic compound that regulates plant growth and development as well as their responses to biotic and abiotic stresses (Khan et al., 2015). The foliar application of SA has been shown to increase grain filling period, TGW, GPS, grain yield, and some other key yield-related traits in wheat genotypes (Karim and Khursheed, 2011; Ziasmin et al., 2017; Singh et al., 2019; Jatana et al., 2021; Stanislawska-Glubiak and Korzeniowska, 2021; Abdi et al., 2022). The foliar application of 0.75 mM SA at 30 days after sowing (DAS) and seed treatment with 0.75 mM SA produced the best results under late sown and timely sown conditions, thereby, significantly increasing grain filling period, grain yield, proline content, and soluble sugar content in leaves, plant height, and tiller number in two wheat varieties (viz., NW5054 and NW2036) when compared to the control (Singh et al., 2019).

In addition to high GPC, *Gpc-B1 is* linked to the yellow rust resistance gene *Yr36*, and excellent gene-based markers are available for monitoring combination of these genes (Uauy et al., 2005). Lastly, pyramiding and introgression of major QTLs/genes for various traits of economic importance via marker-assisted selection (MAS) have been shown to have the positive results in wheat (e.g., Sharma et al., 2021; Gill et al., 2022; Gupta et al., 2022). We at Punjab Agricultural University (PAU), Ludhiana, India have successfully used MAS for GPC enrichment in our wheat breeding programs, pyramiding the *Gpc-B1* gene and the stripe rust resistance gene *Yr15* in the background of a popular wheat cultivar “PBW550” using a unique combination of multi-disciplinary and multi-pronged approaches (e.g., Tanin et al., 2022b). This germplasm has higher GPC levels, which is important for reducing mass malnutrition and hunger. Consumers who are most affected by rising food prices will be the primary beneficiaries, ultimately improving food security and sustainable production.

The grain yield and GPC have been reported to have a negative correlation, which makes it difficult to increase GPC without sacrificing yield, developing high GPC wheat genotypes unlikely to be commercially successful due to yield penalty. This situation necessitates a detailed examination of how the wheat genotypes achieve high GPC, as well as a simple method of using economically feasible intervention(s) to compensate the yield losses associated with high GPC. To achieve the above-mentioned objective, the present study was planned to investigate the effects of foliar applications of PN and SA on senescence, grain filling period, yield, and yield components, with a particular emphasis on minimizing the yield penalty associated with the *Gpc-B1* gene in order to combine higher GPC with no yield penalty in an individual wheat genotype, combining both higher grain yield and increased GPC.

## Materials and methods

A breeding program was previously initiated in the Department of Plant Breeding and Genetics, Punjab Agricultural University (PAU), to generate a set of near isogenic lines (NILs) with a special focus on transferring the high GPC loci *Gpc-B1* from a hard red spring wheat cultivar ‘GLUPRO’ into the genetic background of an elite wheat variety (PBW550). The wheat cultivar PBW550, widely known for its desirable yield and quality characteristics, was developed in PAU and released to be grown under irrigated conditions in the Northern Western Plains Zone, India. The current study employed nine NILs containing the *Gpc-B1* gene and three commercial wheat varieties lacking the *Gpc-B1* gene (**Table 1**). The successful introgression of high GPC loci into the backgrounds of all nine NILs was confirmed using a *Gpc-B1*-specific KASP marker (data not provided) (Tanin et al., 2022b).

**Table 1:** Wheat genotypes (nine NILs, and three elite varieties) evaluated in the current investigation.

Serial No.	Genotypes	*Gpc-B1*	Pedigree
1	BWL6964	+	PBW550//*Yr15*/6*Avocet/3/2*PBW550/4/GLUPRO/3*PBW568//3*PBW550
2	BWL7503	+	PBW550//*Yr15*/6*Avocet/3/2*PBW550/4/GLUPRO/3*PBW568//3*PBW550
3	BWL7504	+	PBW550//*Yr15*/6*Avocet/3/2*PBW550/4/GLUPRO/3*PBW568//3*PBW550
4	BWL7506	+	PBW550//*Yr15*/6*Avocet/3/2*PBW550/4/GLUPRO/3*PBW568//3*PBW550
5	BWL7507	+	PBW550//*Yr15*/6*Avocet/3/2*PBW550/4/GLUPRO/3*PBW568//3*PBW550
6	BWL7508	+	PBW550//*Yr15*/6*Avocet/3/2*PBW550/4/GLUPRO/3*PBW568//3*PBW550
7	BWL7509	+	PBW550//*Yr15*/6*Avocet/3/2*PBW550/4/GLUPRO/3*PBW568//3*PBW550
8	BWL7510	+	PBW550//*Yr15*/6*Avocet/3/2*PBW550/4/GLUPRO/3*PBW568//3*PBW550
9	BWL7511	+	PBW550//*Yr15*/6*Avocet/3/2*PBW550/4/GLUPRO/3*PBW568//3*PBW550
10	PBW761	–	PBW550//*Yr15*/6*Avocet/3/2*PBW550
11	PBW725	–	PBW621//GLUPRO/3*PBW568/3/PBW621
12	HD3086	–	DBW14/HD 2733//HUW 468

+ confers the presence of Gpc-B1 gene, while – confers the absence of Gpc-B1 gene.

### Field management and agronomic practices

To study the effect of PN and SA on GPC, senescence and key yield related traits in wheat, two independent field experiments were conducted during the main season 2020-21 in the wheat experimental area of the Department of Plant Breeding and Genetics, PAU, Punjab, India. The experimental area is located at an altitude of 247 meters above the mean sea level at a latitude of N and a longitude of 75˚48´E and, therefore, experiences a sub-tropical and semi-arid climate with cold (November to January) and mild winters (February and March), and also hot and dry summer (April to July). Both PN (@2%) and SA (@75 ppm) were sprayed twice (first spray at booting stage and second spray at anthesis stage) on the wheat genotypes grown in different field experiments. Concentrations of PN and SA tested and developmental stages targeted were selected based on a thorough survey of the earlier published studies and preliminary experiments (data not shown) conducted earlier by us. A total of 2 mg of PN powder was dissolved in 100 ml of distilled water to obtain a 2% potassium nitrate solution. Similarly, 50 mg of standard SA was dissolved in 95% alcohol in a volumetric flask to dilution formula from the ratio between the concentration and the volume (Saputra et al., 2022). Both PN and SA were sprayed on leaves at an equal rate of 100 cc per m^2^ to every genotype/plot (540 cc per plot/genotype) using a backpack CO_2_-based pressure sprayer. For the control treatments, PN and SA were replaced with distilled water.

In both the experiments, the environmental conditions (including rainfall, temperature, and humidity) differed significantly (**Supplementary Table 1**). For each chemical, the experiment was conducted in three replications using a randomized block design (RBD). Each genotype was grown at the standard rate of 100 kg seed per hectare recommended by PAU, in a separate plot (plot size of 5.4 m^2^, six rows of 4.5 m long with a row-to-row distance of 20 cm). In case of the PN and SA experiments, the trials were sown on November 22 and November 26, 2020, respectively. During the growing season, the standard field management practices were applied according to PAU recommendations (online available at https://www.pau.edu/content/ccil/pf/pp_rabi.pdf) for raising the crops. For better crop growth, a PAU-recommended package of NPK fertilizers (N = 50 kg/acre, P = 25 kg P_2_O_5_/acre, and K = 12 kg K_2_O/acre) was applied. Furthermore, manual weeding and chemical weed control were implemented before and during the growing season inside and across the surrounding border areas of the fields. In the experimental fields, standard methods to control insect-pests and diseases were also used when required.

### Data recorded on different traits

Our study mainly aimed to investigate the effects of PN application on a range of agronomic and physiological traits. Additionally, a preliminary experiment was conducted to assess the effects of SA application on some selected agronomic traits. In the experiments conducted for the evaluation of the effect of foliar applications of PN and SA, data on number of days to flowering (DTF), and number of days to maturity (DTM) was recorded when 75% of the plants in a plot were in that stage. Furthermore, a set of five plants was randomly selected in each plot for recording the data on following traits- plant height (PH), number of spikelets per plant (SPS), number of grains per spike (GPS), 1000-grain weight (TGW), and grain protein content (GPC) as described in one of our earlier studies (Tanin et al., 2022b). The yield per plot (YPP) was recorded by weighing the total wheat grains harvested from each plot. In addition to the traits listed above, the data on following physiological traits was also recorded during the experimental trial conducted to evaluate the effect of PN application-

#### (i) Chlorophyll content in flag leaf

A total of 10 plants were tagged in each plot. Consequently, the chlorophyll content was measured on the flag leaf of each individual plant using a hand-held chlorophyll concentration meter (MC-100, Utah 84321, USA). The data for each genotype was recorded at booting stage (Chl. BS), anthesis stage (Chl. AS), 10, 20, 25, and 30 days after anthesis (Chl. 10DAA, Chl. 20DAA, Chl. 25DAA, and Chl. 30DAA). In each replication, the value of each genotype consisted of the average of 10 flag leaf samples (3 readings per flag leaf). The experimental error was decreased as a result of the large number of samples that were collected for the determination of the chlorophyll content. The chlorophyll content reading was recorded as chlorophyll content index (CCI).

#### (ii) Senescence in peduncle

To measure the senescence in wheat peduncles, 10 peduncles were randomly tagged from each plot, and the percentage of peduncles completely turned yellow at 30 DAA (YP 30DAA) was determined by counting the number of yellow peduncles from these randomly selected peduncles. The readings for the yellow peduncles were recorded in percentage (%).

#### (iii) Flag leaf nitrogen content

For measuring the leaf nitrogen content, five wheat plants were randomly tagged in each plot, and subsequently flag leaf samples from each tagged plant were collected (five samples per plot), packed in envelops, and dried using an oven. The sampling dates for flag leaf nitrogen content analysis were similar to those for chlorophyll content measurement viz., flag leaf nitrogen content analysis at booting stage (N BS), anthesis stage (N AS), 10, 20, 25, and 30 days after anthesis (N 10DAA, N 20DAA, N 25DAA, and N 30DAA, respectively). Later on, the flag leaf samples were subjected to nitrogen content analysis following the Kjeldahl’s distillation method as described by (Piper, 2019). About 0.5 g of dried grinded leaf samples were mixed with a pinch of digestion mixture of potassium sulphate (K_2_SO_4_), copper sulphate (CuSO_4_), selenium powder, and mercury oxide for this purpose, and then digested in 10 ml of concentrated sulphuric acid (H_2_SO_4_). All of the chemical mixture was placed in the digestion tube and left overnight before being heated in Kjeldahl’s digestion unit from 75^0^C to 375^0^C for 2.5 hour/each sample during the next day to digest the colourless content. The digestion mixture was then used for distillation by filling a volumetric flask to 50 ml with distilled water. Following that, the final titration was performed, and the nitrogen content (per leaf sample) was calculated using the following equation given by Islam et al. (2003):


$$\text{N}(\%)=\frac{14\;\times Normality\;of\;Acid\;\times Titration\;Volume-Blank\;Value}{Sample\;Weight\;\times 10}$$

#### (iv) Measurements of grain nitrogen content and straw nitrogen content

Grain nitrogen content (GNC; %) and straw nitrogen content (SNC; %) were determined following the Kjeldahl’s distillation method as discussed above for the estimation of nitrogen content in flag leaves. In this method, half a gram of completely dried and grinded grain and straw samples were mixed with a small amount of digestion mixture and later digested in 10 ml of H_2_SO_4_ for 150 minutes per sample. The digestion tubes containing the chemical mixture were kept overnight at room temperature and then heated in the digestion unit for 150 minutes at a temperature of up to 375°C to digest the content. The mixture was later subjected to a distillation process following the final titration, and the nitrogen content (per grain sample and straw sample) was calculated using the above-mentioned equation.

### Statistical analysis

The experiments were based on randomized block designs. The pooled analysis of variance (ANOVA) was performed for the data collected from each experiment, using the agricolae package of RStudio software version 4.0.3. The least significant difference (LSD) test was utilized in the context of the ANOVA when the F-ratio suggested the rejection of the null hypothesis H_0_, that is, when the difference between the treatments and genotypes means were significant. This test was useful in identifying treatments with statistically different means.

## Results

### Experiments conducted for the evaluation of the effect of foliar application of SA

Based on the data recorded in experimental trial conducted to evaluate the effect of SA, a combined ANOVA was carried out to determine the effects of the genotypes and the treatments that were tested in this study. The combined ANOVA involving all the eight traits is provided in **Supplementary Table 2**. The variation due to genotype (G) was highly significant for all the eight traits at a 0.001% level of significance, while the variation due to SA treatment (T) was found to be significant at different levels of significance, for instance, DTM (0.001%), GPS (0.001%), TGW (0.01%), and YPP (0.001%). Overall, in this experiment, a significant high level of variation was observed among the wheat genotypes in terms of GPC and yield-related traits.

### Effect of SA on yield and related traits in advanced wheat genotypes

The data provided in **Table 2** represents the significant level of variations due to the SA treatment among the genotypes. Foliar application of SA significantly increased the DTM by an average of 2.31 days as compared to the control. Among the genotypes, HD3086 recorded the highest DTM (129.33 days), whereas the lowest DTM was observed in BWL7511 (122 days). The genotype, PBW725, recorded the highest increase in DTM (3.33 days) with the SA spray over the control. The increase in grain filling period due to SA application was higher in the genotypes without *Gpc-B1* as compared with the genotypes with *Gpc-B1* gene. The GPS was significantly increased by an average of 3.17 grains due to foliar spray of SA in all the genotypes under study. PBW725 had the most GPS (63 grains), which was significantly higher than all other genotypes tested during the experiment. BWL7507 had the lowest GPS (45 grains), which was significantly lower than all other genotypes. The greatest increase in GPS was observed in PBW725 (6 grains) with SA application when compared to the control. When compared to the control, the lowest GPS improvement was observed in BWL6964 (1.34 grains) with SA spray.

**Table 2:** Effects of SA treatment on yield component compared to the control.

	GPS		DTM		TGW		YPP	
Genotypes	SA	Control	SA	Control	SA	Control	SA	Control
**BWL6964**	48.67 ± 0.58	47.33 ± 0.58	122.67 ± 0.58	120.33 ± 0.58	28.05 ± 0.05	27.5 ± 0.50	2.19 ± 0.03	2.16 ± 0
**BWL7503**	51 ± 1	47.67 ± 0.58	124.33 ± 0.58	122.33 ± 0.58	29.65 ± 1.55	29.43 ± 1.59	2.6 ± 0.01	2.47 ± 0.01
**BWL7504**	51 ± 1	49 ± 0	123.33 ± 0.58	121.33 ± 0.58	30 ± 1	29.33 ± 1.53	3.03 ± 0.06	2.84 ± 0.08
**BWL7506**	60 ± 1	55 ± 0	123.67 ± 0.58	121.33 ± 0.58	33.7 ± 3.10	31.93 ± 3.27	3.51 ± 0.03	3.24 ± 0
**BWL7507**	45 ± 1	44.67 ± 0.58	123 ± 0	121.33 ± 0.58	32.35 ± 1.65	31.57 ± 1.25	2.03 ± 0.03	2.01 ± 0.02
**BWL7508**	59 ± 1	54.67 ± 0.58	125 ± 0	122.33 ± 0.58	32.85 ± 0.35	31.25 ± 0.72	3.41 ± 0.01	3.2 ± 0.03
**BWL7509**	48 ± 1	45.67 ± 0.58	124 ± 0	122.33 ± 0.58	33.5 ± 0.40	33.17 ± 0.67	2.26 ± 0.02	2.24 ± 0.03
**BWL7510**	54.67 ± 0.58	52 ± 0	123.33 ± 0.58	121.33 ± 0.58	32.25 ± 0.95	31.93 ± 1.01	2.79 ± 0.03	2.72 ± 0.03
**BWL7511**	62 ± 1	58.33 ± 0.58	122 ± 1	120.33 ± 0.58	33.3 ± 1.30	31.7 ± 0.85	4.08 ± 0.03	3.78 ± 0
**PBW761**	56 ± 1	54.33 ± 0.58	124.33 ± 0.58	121.33 ± 0.58	39 ± 1	37.5 ± 1.32	3.12 ± 0.09	2.79 ± 0.08
**PBW725**	63 ± 1	57 ± 0	129 ± 1	125.67 ± 0.58	40 ± 0	37.67 ± 0.58	4.42 ± 0.02	3.8 ± 0.03
**HD3086**	62 ± 1	56.67 ± 0.58	129.33 ± 0.58	126.33 ± 0.58	36.45 ± 1.05	34.67 ± 0.58	4.29 ± 0.02	3.85 ± 0.01
**Means**	55.03 ± 6.22	51.86 ± 4.78	124.5 ± 2.32	122.19 ± 1.91	33.43 ± 3.58	32.30 ± 3.08	3.14 ± 0.82	2.93 ± 0.65
**LSD 5%**								
**G**	0.82		0.68		1.57		0.04	
**T**	0.34		0.28		0.64		0.01	

DTM, number of days to maturity; GPS, number of grains per spike; TGW, 1000-grain weight; YPP, yield per plot; SA, salicylic acid; T, treatment, and G, genotype.

**Figure 1:**
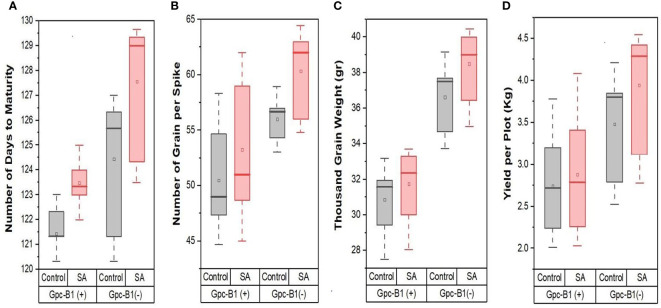
The effects of SA treatment on key agronomic traits such as **(A)** number of days to maturity, **(B)** number of grain per spike, **(C)** thousand grain weight, and **(D)** yield per plot of wheat genotypes with and without the *Gpc-B1* gene.

Furthermore, in the current experiment, all of the tested genotypes conferred a highly significant improvement in GPS with SA spray over their respective control(s). The SA spray on the leaves increased the TGW and YPP significantly, with an average of 1.13 g and 0.21 kg per plot, respectively. PBW725 had the highest TGW (40 g) and yield (4.42 kg per plot), which was significantly higher than all other genotypes for both the traits. PBW725 had the highest levels of enhancement for TGW (2.23 g) and yield (0.62 kg per plot). Similarly, the smallest increases in TGW and YPP were found in BWL7510 (0.32 g), BWL7507 (0.02 kg per plot), and BWL7509 (0.02 kg per plot). Overall, in the light of the results obtained, the foliar application of SA was reported to be extremely successful in delaying the senescence and extending the grain filling period to reduce the yield penalty associated with the *Gpc-B1* gene, and furthermore, the improvement in yield and yield components due to SA spray was higher in the genotypes without *Gpc-B1* as compared to genotypes with the *Gpc-B1* gene. This could be due to the comparatively longer grain filling period with SA application observed in the genotypes which did not carry the *Gpc-B1* gene (**Figure 1**). Furthermore, in the current study, SA foliar spray had no significant effect on DTF, PH, SPS, or GPC.

### Experiment conducted for the evaluation of the effect of foliar application of PN

Based on the data recorded in experimental trial conducted at Ludhiana, a pooled ANOVA was carried out to determine the effects of the genotypes and the treatments that were tested in this study. The pooled ANOVA concerning all the twenty-three traits (which is recorded on different dates) is provided in **Supplementary Table 3**. The variation due to genotype (G) was highly significant for all the traits either at a 0.001% or 0.01% level of significance, while in the case of treatment (T), the variation was highly significant for DTM, GPS, TGW, YPP, Chl. AS, Chl. 10DAA, N BS, N AS, N 10DAA, YP 30DAA, and SNC at 0.001% level of significance. Furthermore, the G x T interaction was also observed as significant for DTF, PH, SPS, GPS, Yield, Chl. BS, Chl. 25DAA, N BS, N AS, N 10DAA, YP 30DAA, and SNC. In the present study, a significant level of differences was observed for all the physiological, and yield-related traits.

### Effect of PN on yield and related traits in advanced wheat genotypes

**Table 3** represents the differences in the values of yield and its components achieved after foliar application of PN. The foliar spray of PN significantly increased the GPS by an average of 6.42 grains in all tested genotypes over their corresponding control. Among the genotypes, BWL7502 recorded the highest number of GPS (61.3 grains), which was significantly higher than all other genotypes, whereas, lowest number of GPS (48.3 grains) was recorded by HD3086. The genotype BWL7502 showed the highest improvement in GPS (14.5 grains) with PN spray as compared to its respective control. Similarly, all the genotypes showed significantly higher GPS with PN spray as compared to the control. The DTM was significantly increased with PN application by an average of 1.03 days over the control in all the genotypes considered in the present study. PBW725 showed the highest DTM (133.67 days), which was significantly higher than all other tested genotypes. The BWL6964 and BWL7502 recorded the lowest DTM (125 days), which was significantly lower than all other genotypes. Among the genotypes, BWL6964 exhibited the highest improvement in DTM (2 days) due to foliar application of PN over the control.

**Table 3:** Comparison of the effects of PN treatment on yield components compared to control.

	GPS		DTM		TGW		YPP	
Genotypes	PN	Control	PN	Control	PN	Control	PN	Control
**BWL6964**	57.4 ± 0.4	49.6 ± 1.91	125 ± 1	123 ± 1	32.68 ± 0.79	30.56 ± 0.5	3.4 ± 0.05	3.35 ± 0.08
**BWL7502**	61.3 ± 1.3	46.8 ± 2	125 ± 1	123.5 ± 0.5	32.67 ± 1.89	31.56 ± 0.33	3.57 ± 0.14	3.5 ± 0.1
**BWL7504**	54 ± 2.56	50.8 ± 2.17	126 ± 0	124.5 ± 0.5	29.94 ± 0.25	28.42 ± 0.5	3.10 ± 0.06	3 ± 0.09
**BWL7506**	50.33 ± 2.66	45 ± 2.23	125.33 ± 0.58	124.5 ± 0.5	36.36 ± 0.58	34.84 ± 0.5	3.34 ± 0.15	3.1 ± 0.05
**BWL7507**	52 ± 2.6	43.6 ± 1.83	126 ± 0	125 ± 0	34.88 ± 1.3	33.02 ± 0.48	3.12 ± 0.07	3 ± 0.08
**BWL7508**	53.3 ± 1.47	50.4 ± 1.91	125.67 ± 0.58	124.5 ± 0.5	32.42 ± 0.63	30.87 ± 0.5	3.16 ± 0.08	3 ± 0.09
**BWL7509**	54.9 ± 2.52	50 ± 2.17	125.33 ± 0.58	124.5 ± 0.5	32.07 ± 0.9	30.28 ± 0.5	3 ± 0.33	2.05 ± 0.09
**BWL7510**	52.3 ± 0.5	46 ± 2.23	125.33 ± 0.58	124.5 ± 0.5	33.25 ± 0.9	31.17 ± 0.17	3.74 ± 0.04	3.55 ± 0.08
**BWL7511**	53.53 ± 2.25	48 ± 1.8	125.33 ± 0.58	124.5 ± 0.5	33.98 ± 1.75	31.54 ± 0.5	3.14 ± 0.11	3 ± 0.05
**PBWL761**	54.2 ± 2.51	50 ± 2.23	125.67 ± 0.58	125.5 ± 0.5	35.68 ± 1.3	33.8 ± 0.5	3.46 ± 0.06	3.4 ± 0.02
**PBW725**	54.3 ± 3.7	45.4 ± 1.87	133.67 ± 0.58	132.5 ± 0.5	39.44 ± 1.51	34.31 ± 0.44	4.13 ± 0.05	4.05 ± 0.1
**HD3086**	48.3 ± 2.5	43.2 ± 1.91	131 ± 1	130.5 ± 0.5	35.09 ± 0.46	34.49 ± 0.5	2.19 ± 0.09	2 ± 0.08
**Means**	53.82 ± 3.29	47.4 ± 2.75	126.61 ± 2.75	125.58 ± 2.87	34.04 ± 2.46	32.07 ± 2	3.28 ± 0.47	3.08 ± 0.59
**LSD 5%**								
**G**	2.46		0.69					
**T**	1		0.28		0.82		0.14	
					0.34		0.06	

DTM, number of days to maturity; GPS, number of grain per spike; TGW, 1000-grain weight; YPP, yield per plot; PN, potassium nitrate; T, treatment, and G, genotype.

**Figure 2:**
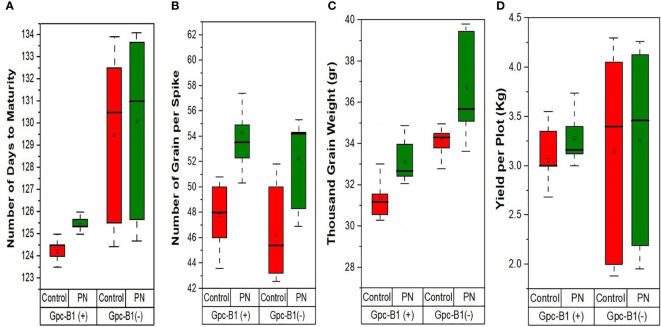
The effects of PN treatment on key agronomic traits such as **(A)** number of days to maturity, **(B)** number of grain per spike, **(C)** thousand grain weight, and **(D)** yield per plot of wheat genotypes with and without the *Gpc-B1* gene.

Similarly, the TGW was significantly improved with the foliar application of PN by an average enhancement of 1.97 g in all the genotypes. The genotype PBW725 conferred the highest TGW (39.44 g), which was significantly higher than all other genotypes evaluated in this study. The lowest TGW was observed in BWL7504 (29.94 g), which was significantly lower than other genotypes. PBW725 also showed the highest enhancement in TGW (5.13 g) with PN application as compared to the control. The PN application on leaves significantly increased the total YPP by an average of 0.2 kg per plot in all the genotypes. The highest YPP (4.13 kg per plot) was recorded by PBW725, which was significantly higher than all other studied genotypes. The lowest YPP (2.19 kg per plot) was observed in HD3086, which was significantly lower as compared to other genotypes. The highest YPP enhancement (0.95 kg per plot) was recorded by BWL7509 with the PN spray over the control. Overall, the enhancement in yield and yield components due to PN foliar application was greater in the genotypes without *Gpc-B1* over the genotypes with the *Gpc-B1* gene (**Figure 2**). This could be due to the comparatively longer grain filling period with SA application observed in the genotypes which did not carry the *Gpc-B1* gene. Compared to the control, the foliar spray of PN had no significant effect on DTF, PH, SPS, and GPC traits.

### Effect of PN on flag leaf chlorophyll content in wheat NILs and cultivars

**Figure 3:**
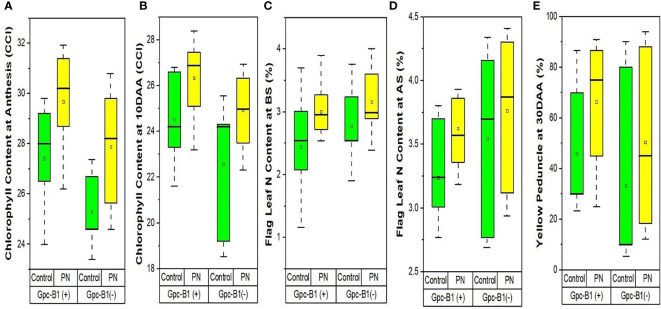
The effects of PN treatment on traits such as **(A)** chlorophyll content at anthesis, **(B)** chlorophyll content at 10DAA, **(C)** flag leaf N content at booting stage, **(D)** flag leaf N content at anthesis stage, and **(E)** yellow peduncle at 30DAA of wheat genotypes with and without the *Gpc-B1* gene.

The **Table 4** represents the differences in flag leaf chlorophyll contents achieved after foliar spray of PN. As clear from the **Table 4**, the foliar spray of PN significantly increased the flag leaf chlorophyll content by an average of 2.35 CCI at AS and 1.96 CCI at 10DAA as compared to the control. Among the genotypes, BWL7510 with 31.93 CCI at AS and BWL7507 with 28.4 CCI at 10DAA showed the highest flag leaf chlorophyll contents, that were significantly higher than all other genotypes evaluated in the current study. The HD3086 recorded the lowest flag leaf chlorophyll content with 25.65 CCI at AS and 23.5 CCI at 10DAA) as compared to other genotypes. The genotypes, BWL7502 with 5.55 CCI at AS, and HD3086 with 4.30 CCI at 10DAA were observed to have the highest improvements in flag leaf chlorophyll contents with the PN application over the control. Furthermore, the foliar application of PN had no significant effects on the flag leaf chlorophyll contents at the BS, 20DAA, 25DAA, and 30DAA, compared to the control. Furthermore, chlorophyll content enhancement due to PN application in *Gpc-B1* positive wheat genotypes was much higher than in *Gpc-B1* negative wheat genotypes (**Figures 3A, B**).

Table 4Comparison of the effects of PN treatment on physiological and other key traits.

**Table:** 

	Chl. AS		Chl. 10DAA		N BS		N AS	
Genotypes	PN	Control	PN	Control	PN	Control	PN	Control
**BWL6964**	30.2 ± 0.6	28.4 ± 1.95	27.45 ± 0.65	24.2 ± 1.65	2.95 ± 0.29	2.08 ± 0.03	3.93 ± 0	2.77 ± 0.08
**BWL7502**	29.55 ± 0.05	24 ± 2	25.4 ± 0.20	23.3 ± 2	2.77 ± 0	2.54 ± 0.10	3.35 ± 0.35	2.77 ± 0.1
**BWL7504**	31.4 ± 0.1	29.2 ± 2.08	27.40 ± 0.53	26.6 ± 1.4	2.95 ± 0.06	2.77 ± 0.08	3.35 ± 0.12	3.01 ± 0.09
**BWL7506**	31.7 ± 0.46	29.8 ± 1.95	28.37 ± 0.12	26.6 ± 2	3.27 ± 0.14	3.01 ± 0.08	3.85 ± 0.05	3.7 ± 0.07
**BWL7507**	30.53 ± 0.21	28 ± 2.23	28.4 ± 0.95	26.8 ± 1.2	3.90 ± 0.05	3.7 ± 0.05	3.88 ± 0.08	3.7 ± 0.07
**BWL7508**	28.7 ± 0.56	26.5 ± 1.5	26.87 ± 0.57	24.1 ± 1.9	3.35 ± 0.13	3.01 ± 0.09	3.86 ± 0.10	3.7 ± 0.05
**BWL7509**	26.2 ± 2	24.9 ± 1.1	23.2 ± 3.9	21.6 ± 1.4	2.54 ± 0.46	1.16 ± 0.04	3.36 ± 0.35	3.01 ± 0.07
**BWL7510**	31.93 ± 0.67	29.4 ± 1.6	24.85 ± 0.35	22.8 ± 1.2	2.72 ± 0.17	2.31 ± 0.09	3.47 ± 0.21	3.24 ± 0.07
**BWL7511**	26.95 ± 0.15	26.5 ± 1.5	25.1 ± 0.3	24.7 ± 1.3	2.6 ± 0.40	1.39 ± 0.06	3.57 ± 0.37	3.24 ± 0.06
**PBWL761**	28.2 ± 1.18	26.7 ± 1.7	24.97 ± 0.59	24.3 ± 1.7	3.60 ± 0.10	3.24 ± 0.06	4.30 ± 0.12	4.16 ± 0.04
**PBW725**	29.8 ± 5.3	24.6 ± 1.5	26.33 ± 0.5	24.2 ± 1.8	2.99 ± 0.01	2.54 ± 0.07	3.87 ± 0.03	3.7 ± 0.05
**HD3086**	25.65 ± 0.45	24.6 ± 2.14	23.5 ± 0.4	19.2 ± 1.8	2.89 ± 0.12	2.54 ± 0.06	3.12 ± 0.12	2.77 ± 0.08
**Means**	29.23 ± 2.13	26.88 ± 2.06	25.99 ± 1.76	24.03 ± 2.19	3.04 ± 0.41	2.52 ± 0.73	3.66 ± 0.34	3.31 ± 0.47
**LSD 5%**								
**G**	1.93		1.55		0.18		0.17	
**T**	0.79		0.63		0.07		0.07

**Table:** 

	N 10DAA		SNC		YP 30DAA			
Genotypes	PN	Control	PN	Control	PN	Control		
**BWL6964**	2.70 ± 0.08	2.54 ± 0.06	0.69 ± 0.23	0.23 ± 0.01	90 ± 10	40 ± 20		
**BWL7502**	2.89 ± 0.35	2.08 ± 0.10	0.46 ± 0	0.23 ± 0.05	86.67 ± 11.55	80 ± 10		
**BWL7504**	2.77 ± 0.23	2.54 ± 0.06	0.79 ± 0.06	0.69 ± 0.01	91 ± 3.61	86.67 ± 5.77		
**BWL7506**	3.45 ± 0.08	3.24 ± 0.03	0.29 ± 0.03	0.23 ± 0.02	85 ± 15	23.33 ± 15.28		
**BWL7507**	2.94 ± 0.09	2.77 ± 0.06	0.35 ± 0.12	0.23 ± 0.01	60 ± 10	30 ± 20		
**BWL7508**	2.91 ± 0.06	2.77 ± 0.06	0.58 ± 0.12	0.23 ± 0.01	45 ± 15	30 ± 20		
**BWL7509**	2.78 ± 0.46	2.31 ± 0.07	0.32 ± 0.06	0.23 ± 0.02	75 ± 25	70 ± 10		
**BWL7510**	2.43 ± 0.11	2.31 ± 0.06	0.35 ± 0.12	0.23 ± 0.01	25 ± 15	23.33 ± 15.28		
**BWL7511**	2.79 ± 0.01	1.85 ± 0.05	0.35 ± 0.12	0.23 ± 0.01	40 ± 30	30 ± 20		
**PBW761**	2.54 ± 0.69	2.31 ± 0.06	0.35 ± 0.12	0.23 ± 0.02	18.33 ± 7.64	10 ± 10		
**PBW725**	3 ± 0.23	2.54 ± 0.06	0.37 ± 0.09	0.23 ± 0.01	45 ± 35	10 ± 10		
**HD3086**	2.52 ± 0.03	2.31 ± 0.02	0.36 ± 0.02	0.23 ± 0.02	88 ± 2.65	80 ± 10		
**Means**	2.81 ± 0.27	2.46 ± 0.36	0.44 ± 0.16	0.27 ± 0.13	61.86 ± 26.4	42.78 ± 28.35		
**LSD 5%**								
**G**	0.24		0.09		19.19			
**T**	0.1		0.04		7.83			

Chl. AS, chlorophyll content at anthesis stage; Chl. 10DAA, chlorophyll content at 10 days after anthesis; N BS, nitrogen content at booting stage; N AS, nitrogen content at anthesis stage; N 10DAA, nitrogen content at 10 days after anthesis; YP 30DAA, yellow peduncle at 30 days after anthesis; SNC, straw nitrogen content, PN, potassium nitrate; T, treatment, and G, genotype.

### Effect of PN on flag leaf nitrogen content, straw nitrogen content and peduncle senescence in wheat NILs and cultivars

The **Table 4** contains the data revealing the differences for nitrogen contents in flag leaf and straw, and senescence in peduncles among the genotypes achieved after treating with PN. The flag leaf nitrogen content was significantly increased with the foliar spray of PN by an average of 0.52% at BS, and 0.35% at both AS and 10DAA in the evaluated genotypes. The highest flag leaf nitrogen contents were observed in BWL7507 (3.9% at BS), PBW761 (4.3% at AS), and BWL7506 (3.45% at 10DAA), which were significantly higher than all other genotypes in each developmental stage. The lowest flag leaf nitrogen content was recorded by BWL7509 with 2.54% at BS, followed by HD3086 with 3.12% at AS, and BWL7510 with 2.43% at 10DAA which were significantly lower as compared to other genotypes at each developmental stage. The highest flag leaf nitrogen enhancements with PN was observed in BWL7509 with 1.38% at BS, followed by BWL6964 with 1.16% at AS, and BWL7511 with 0.94% at 10DAA after the PN spray as compared to the control. As a result, the flag leaf nitrogen content was higher in genotypes with no *Gpc-B1* gene compared to the genotypes carrying the *Gpc-B1* gene at BS and AS (**Figures 3C, D**).

The foliar application of PN significantly increased the SNC by an average of 0.17% over the control. BWL7504 was observed to have the highest SNC with 0.79%, which was significantly higher than all other genotypes. The lowest SNC was recorded by BWL7506 with 0.29%, which was statistically similar to BWL7507, BWL7509, BWL7510, BWL7511, and PBW761 but significantly lower than the remaining genotypes. The genotype BWL6964 recorded the highest enhancement in SNC (0.46%) with PN over the control. The YP 30DAA was significantly increased by an average of 19.08% due to foliar application of PN in all the genotypes as compared to the control. The highest YP 30DAA was observed in BWL7504 (91%) which was statistically similar to BWL6964, BWL7502, BWL7506, and HD3086 but significantly higher than all other genotypes. The lowest YP 30DAA was recorded by PBW761 (18.33%) which was significantly lower than all other genotypes. The highest enhancement of YP 30DAA with PN was recorded by BWL7506 (61.67%) as compared to the control. Overall, the rate of senescence in peduncle was much higher in *Gpc-B1* negative genotypes than in *Gpc-B1* positive wheat genotypes (**Figure 3E**). However, the foliar spray of PN had no significant effects on N 20DAA, N 25DAA, N 30DAA, and GNC traits.

## Discussion

In wheat, the GPC is considered the most important factor affecting nutritional quality. Grain protein, therefore, has significant potential to increase economic value of wheat grains (Gudi et al., 2022a). *Gpc-B1* is the major regulator of GPC in wheat, which is responsible for more than 60% of phenotypic variation for the protein content in grains (Uauy et al., 2006; Saini et al., 2020). It is commonly known that higher GPC is negatively associated with grain yield in wheat, thus hampering the breeding efforts aimed at simultaneous improvement of GPC and yield in wheat genotypes (Tanin et al., 2022b). In addition to significant effects on GPC, the *Gpc-B1* is associated with the earlier initiation of senescence, shorter grain filling period, higher percentage of yellow peduncle, and more efficient nitrogen remobilization in wheat (Uauy et al., 2006). The SA and PN are known to play significant roles in delaying the leaf senescence and improving tolerance against biotic and abiotic stresses (El-Tayeb, 2005; Ibrahim et al., 2014; Jatana et al., 2021; Gudi et al., 2022b; Tanin et al., 2022a).

Application of SA on leaves significantly affects the wheat physiology, including seed germination, normal growth, yield production, nutrient uptake and remobilization, photosynthesis efficiency, stomatal density and transpiration rate, root development, and biomass enhancement (Azmat et al., 2020; Dias et al., 2021; Wang et al., 2021);. Under stress conditions, SA regulates crucial plant physiological processes, including photosynthesis, proline metabolism, nitrogen metabolism, glycine-betaine production, antioxidant defence system (generation of reactive oxygen species), and plant-water relations, and thus protects plants from abiotic stresses (reviewed in Khan et al., 2015). Furthermore, the SA has been shown to play an important role in the regulation of leaf senescence via its effects on lipid metabolism (Xiao et al., 2010), autophagy (Yoshimoto et al., 2009), and the generation of reactive oxygen species (Guo et al., 2017). Apart from influencing senescence progression, SA influences senescence initiation by simultaneously inducing both positive and negative senescence regulators (Besseau et al., 2012). Grain yield enhancement was observed in wheat due to foliar spray of SA (Safar-Noori et al., 2018). Similarly, the PN regulates different biochemical and physiological processes that influence plant growth and metabolism, including seed germination and emergence, stomatal regulation, phloem transport, cation-anion balance, photosynthesis, energy transfer and contributes to the survival of plants exposed to various abiotic stresses (Wang et al., 2013). In this study, nine NILs carrying the *Gpc-B1* gene and three elite wheat varieties with no *Gpc-B1* gene were used to test the effects of foliar application of SA and PN on different yield-related traits, nitrogen concentrations in different tissues and some key physiological parameters. Previously, different concentrations of SA and PN were used to delay senescence and extend the grain filling period in cereal crops, including wheat, in order to increase grain yield (Singh et al., 2019; Singh and Singh, 2020; Jatana et al., 2021; Singh et al., 2021; Abdi et al., 2022).

The foliar spray of SA significantly increased the number of DTM in the current study, extending the grain filling period in all the evaluated genotypes that resulted into the increased grain yield. These results were consistent with studies earlier conducted in wheat (Singh et al., 2019; Jatana et al., 2021). It is observed that the SA application supports and further improves the photosynthetic mechanism of the wheat crop (Zheng et al., 2013). In addition, Fan et al. (2022) reported that foliar spray of SA significantly abated the negative effect of high temperature on flag leaf area in wheat and therefore the potential of flag leaf for detecting the light energy remain stable, thus the senescence is delayed in wheat flag leaves. Further, the carotenoids and chlorophyll are the important pigments produced during photosynthesis in plants, and chlorophyll is the major regulator of light energy-based physiological processes. The exogenous application of SA preserves the normal production of chlorophyll content in flag leaves under heat stress and further improves the photosynthetic efficiency and chlorophyll production in wheat flag leaves as a result of involvement of SA in preserving the structure of the chloroplast membrane and maintaining the optimum balance of the antioxidant mechanism in plant chloroplasts (Fan et al., 2022).

Furthermore, the foliar application of SA significantly enhanced the Fv/Fm of flag leaves in wheat, and therefore, due to this improvement, the photosynthetic potential and efficiency of light energy utilization were significantly increased, paving the way for better storage of the photosynthetic outcomes and delaying senescence, thus keeping the photosynthetic stability normal (Aldesuquy et al., 2018; Fan et al., 2022). Gonzalez-Bayon et al. (2019) observed that reduction of SA level in an *Arabidopsis thaliana* accession through introduction of a bacterial gene (i.e., *NahG*) resulted in plant size enhancement after 3 weeks of growth. The *TL1 BINDING TRANSCRIPTION FACTOR1* is an important gene regulating the SA-induced balance among plant’s defense and growth pathways (Smith, 2019), and its expression levels were significantly suppressed in *Arabidopsis* F1s (derived from C24 x Ler) and C24 *NahG* individuals, which may be the reason for growth enhancement. Similarly, the down-regulation of several genes (e.g., *WRKY53*, *ORESARA1*, and *NAC-CONTAINING PROTEIN 29*) associated with SA-induced chlorophyll degradation (senescence) in these individuals could be the regulator of the delayed senescence, thereby causing a longer period of photosynthesis in leaves.

In wheat, the *NAC-S* gene has been reported to confer positive interaction with GPC and delayed senescence with no yield penalty (Sultana et al., 2021). Furthermore, SA spray on wheat leaves also significantly improved the yield components, including GPS and TGW, in all the twelve genotypes tested in the present study, which is in agreement to the previous studies conducted in wheat (Karim and Khursheed, 2011; Dias et al., 2021). The enhancement in yield components with SA application on leaves could be attributed to changes in wheat physiological mechanisms such as influencing the antioxidant defense system (Hasanuzzaman et al., 2019), ion transportation (Simaei et al., 2012), flowering time (Wada and Takeno, 2010), and photosynthesis efficiency (Fariduddin et al., 2003). Further, earlier studies on maize (Ahmad et al., 2018), rice (Jini and Joseph, 2017), barley (Pirasteh-Anosheh et al., 2022), and *Brassica* (Muhal and Solanki, 2016) supports our findings regarding improvements in yield component traits as a result of SA application. The grain yield enhancement with SA spray may be due to higher chlorophyll content, TGW, GPS, and longer grain filling period. The wheat genotype “Prodi” also showed higher grain yield with SA application as compared to the control (Ziasmin et al., 2017). In a more recent study, SA (1.5 mM; once every two days) and NaCl (100 mM, 200 mM; once a week) were sprayed on leaves starting from tillering stage until the complete spike formation stage, resulting a significant increase in shoot weight, root weight, GPC, yield and its components in two wheat varieties (viz., PAN3476, and SST806) (Abdi et al., 2022). In this study, the SA in combination with 100 mM NaCl showed greater enhancement than the SA in combination with 200 mM NaCl.

The foliar application of PN significantly increased the yield and its components, flag leaf chlorophyll content at anthesis and 10DAA, flag leaf nitrogen content at booting stage, anthesis and 10DAA, SNC, and yellow peduncle colour at 30DAA. The number of DTM was significantly increased by an average of more than one day with PN spray over the control. A similar increase in DTM with PN spray was also observed in earlier studies in wheat (Singh et al., 2013; Devi et al., 2017; Agrawal et al., 2021). Therefore, senescence is delayed and the grain filling duration is extended, thus improving key yield-related traits. In wheat, yield is a highly complex trait regulated by a number of genes and is determined by two major attributes, viz., TGW and the GPS (Yadav et al., 2021; Saini et al., 2022a). The grain yield improvement due to foliar application of PN could be due to a longer grain filling period, higher TGW, and GPS as compared to the control in wheat, the GPS is measured by integrating both the number of spikelets per spike and the number of grains per spikelet. Wheat spikelets carry more than a single grain, unlike the spikelets of other cereal crops such as rice and barely (Zhou et al., 2021; Singh R. et al., 2022). Thus, wheat spikelet is one of the most important grain yield attributes.

The capacity for GPC in wheat is significantly affected by the spike architecture. Wheat is known to have an unbranched inflorescence. Furthermore, spikelets are placed along the primary axis of inflorescence (Koppolu and Schnurbusch, 2019). Since a number of florets are available on rachilla to establish the spikelet, the GPS is finally determined by the number of spikelets having the higher number of fertile florets (Li et al., 2021). The increase in the GPS with PN application may also be due to the longer length of spike and the higher number of grains per spikelet. A similar increase in GPS with the PN application was reported in wheat by Singh and Singh (2020). Additionally, higher grain yield along with the favourable physiological, phenological, and biochemical characteristics were observed with foliar spray of thio-urea at a rate of 500 ppm under drought and irrigated conditions in two wheat genotypes viz., K1006, and K307 (Yadav et al., 2019). Similarly, a number of studies have found that PN spray can significantly improve yield and its components in different crops, including rice (Ali et al., 2021), sorghum (Ávila et al., 2022), maize (Ahmad et al., 2022), and coriander (Elhindi et al., 2016).

Flag leaf chlorophyll content is known for its role as one of the most essential determinants in wheat growth and development (Zhang et al., 2016). The higher rate of chlorophyll degradation and accelerated senescence are correlated with the higher GPC and yield penalty in wheat genotypes (Kade et al., 2005). Foliar application of PN at booting and anthesis stages significantly increased the flag leaf chlorophyll content at anthesis and 10DAA. In addition, at both the stages, the chlorophyll content improvement with PN spray was comparatively higher in wheat genotypes with no *Gpc-B1* gene as compared to the genotypes carrying the *Gpc-B1* gene (**Figures 3A, B**). It is assumed that the lower improvement in flag leaf chlorophyll content with PN application on the leaves is associated with the *Gpc-B1* gene. Further, the improvement in chlorophyll content in genotypes with no *Gpc-B1* gene was comparatively higher during 10DAA as compared to the anthesis stage. However, the expression of *Gpc-B1* in leaves is initiated before anthesis (Uauy et al., 2006). Because the *Gpc-B1* gene starts to express after anthesis, genotypes carrying it may experience faster chlorophyll degradation at 10DAA (Uauy et al., 2006).

During the pre-anthesis period, the nitrogen is provisionally stored in flag leaf, and later on, after anthesis is initiated, it is remobilized from flag leaf to developing grains (Distelfeld et al., 2014). The efficient remobilization of nitrogen from flag leaf into grains is the key factor which significantly determines the protein accumulation in wheat grains. In this study, the application of PN significantly increased the flag leaf nitrogen content at booting, anthesis, and 10DAA. The lower nitrogen content and higher chlorophyll content in flag leaf at anthesis in genotypes carrying the *Gpc-B1* as compared to the genotypes with no *Gpc-B1* (**Figures 3C, D**) provides evidence that *Gpc-B1* gene becomes active in leaves before the grain formation. A similar result previously reported by Kade et al. (2005) support the findings of the present study. In addition, the initiation of *Gpc-B1* expression in leaves after grain formation further suggests a significant role of *Gpc-B1* on nitrogen accumulation in grain at source (leaves) over the sink (grain). The higher rate of senescence enhancement in peduncles could be a combined result of PN spray and high GPC gene in genotypes carrying the *Gpc-B1* gene compared to the genotypes with no *Gpc-B1* gene (**Figure 3E**). In addition, we observed that the pleiotropic effect of the *Gpc-B1* gene on various traits, such as peduncle senescence rate, grain size, and yield, was inconsistent following PN application.

To improve wheat performance in the field, physiological interventions (use of SA and PN) and genetics still need to be combined. In the present study, to mitigate the yield penalty associated with the *Gpc-B1* gene in our germplasm, we sprayed SA and PN on leaves to improve the wheat potential (genetics) aimed to produce a higher grain yield with no negative effect on GPC. The results indicated that the foliar application of SA and PN was an extremely efficient approach to extend the grain filling period and enhance the yield components (TGW and GPS) in wheat lines introgressed with the Gp*c-B1* gene. The comparative result indicates that foliar application of SA was more effective to mitigate the yield penalty associated with the *Gpc-B1* gene as compared with the result obtained with the PN spray. The DTM and YPP were significantly improved by 2.31 days and 0.21 kg/plot, respectively, due to the foliar application of SA, while the PN spray increased the DTM and YPP by 1.03 days and 0.2 kg/plot, respectively. Furthermore, both the SA and PN did not show any significant influence on GPC. In addition, a rapid and more efficient photosynthesis was also observed due to the application of PN which subsequently resulted in higher chlorophyll production in flag leaves. Similarly, physiological intervention and genetics could be more effective to improve the yield potential of wheat when further supported by agronomic intervention (e.g., appropriate date of sowing) (Jatana et al., 2021).

## Conclusions

The results of the present investigation provided evidence that the yield reduction due to the presence of the *Gpc-B1* gene could be compensated through the foliar application of SA and PN in wheat. As a result of the spraying of growth regulators on leaves, wheat growth and development were enhanced. The dose and time (stages) of spraying of growth regulators (PN: 2% at booting and anthesis stages; SA: 75 ppm at booting and anthesis stages) used in present experiment is strongly recommended to mitigate the early senescence caused by the *Gpc-B1* gene. This yield improvement in tested wheat genotypes was caused by growth regulator-mediated longer grain filling period. Furthermore, we showed that the application of SA and PN favorably modulated grain yield and other important traits. Future research might explore how these two chemicals interact with the expression and activity of the gene *Gpc-B1*. The preliminary experiment with SA focused on a limited number of agronomic traits; therefore, future experiments can be designed to investigate the effects of SA on additional key agronomic and physiological traits. More research is needed to determine the exact role of SA and PN sprays in the performance of wheat genotypes with the *Gpc-B1* gene in field experiments.

## Data availability statement

The original contributions presented in the study are included in the article/**Supplementary Materials**. Further inquiries can be directed to the corresponding authors.

## Author contributions

AS, HR, VSS, PS, GSM and SS conceptualized the study, MJT, SG, PK, and PG performed the phenotyping and recorded the original data. MJT analyzed the data. MJT and AS wrote the original draft. DKS helped MJT during this research and further extended his support for improvement of the original draft. All authors contributed to the article and approved the submitted version.
