# Supplementary material for: Application of potassium nitrate and salicylic acid improves grain yield and related traits by delaying leaf senescence in Gpc-B1 carrying advanced wheat genotypes
Source: Front Plant Sci. 2023 Jul 17;14:1107705. doi: 10.3389/fpls.2023.1107705 (PMC10389087; doi:10.3389/fpls.2023.1107705)
Supplement: Supplementary file 1 [file Table_1.doc]

Supplementary Material

**Application of salicylic acid and potassium nitrate improves grain yield and related traits by delaying leaf senescence in *Gpc-B1* carrying advanced wheat genotypes**

**Mohammad Jafar Tanin****^1*^, Achla Sharma****^1*^, Hari Ram^1^, Satinder Singh^1^, Puja Srivastava^1^, G S Mavi^1^, Dinesh Kumar Saini^1^, Santosh Gudi^1^, Pradeep Kumar^1^, Prinka Goyal and V S Sohu^1^**

^1^Department of Plant Breeding and Genetics, Punjab Agricultural University, Ludhiana, Punjab, India

*** Correspondence:** Mohammad Jafar Tanin: [jafartanin@gmail.com](mailto:jafartanin@gmail.com)

Achla Sharma: [achla@pau.edu](mailto:achla@pau.edu)

**Supplementary Table 1:** Brief summary of climatic conditions at the experimental fields (Ludhiana) during 2020-2021

| **Pincode** | **Location** | **Latitude** | **Longitude** | **Altitude** | **Year** | **Env. Condition** | **Nov.** | **Dec.** | **Jan.** | **Feb.** | **Mar.** | **Apr.** |
| --- | --- | --- | --- | --- | --- | --- | --- | --- | --- | --- | --- | --- |
| 141001 | Ludhiana | 30˚N | 75˚E | 247 m | 2020-21 | Temperature | 9-31˚C | 4-26˚C | 4-24˚C | 6-33˚C | 13-35˚C | 14-40˚C |
|  |  |  |  |  |  | Rainfall | 16.6 mm | 6 mm | 11 mm | 17 mm | 5 mm | 14.3 mm |
|  |  |  |  |  |  | Humidity | 27-100% | 39-100% | 39-100% | 31-100% | 27-100% | 13-100% |

Source: https://timeanddate.com/weather/india/

**Supplementary Table 2:** Pooled analysis of variances for yield components of 12 wheat genotypes under SA treatment.

|  |  | **DTF** | | **DTM** | | **SPS** | | **GPS** | |
| --- | --- | --- | --- | --- | --- | --- | --- | --- | --- |
|  | **DF** | **SS** | **MS** | **SS** | **MS** | **SS** | **MS** | **SS** | **MS** |
| **Treatment** | 1 | 0.7 | 0.68 | 95.68 | 95.68^***^ | 0.005 | 0.005 | 180.5 | 180.50^***^ |
| **Genotype** | 11 | 564.8 | 51.35^***^ | 292.49 | 26.59^***^ | 18.866 | 1.715^***^ | 1980.1 | 180.01^***^ |
| **T x G** | 11 | 1.2 | 0.1 | 5.49 | 0.5 | 0.015 | 0.0014 | 50.5 | 4.59^***^ |
|  |  | **PH** | | **TGW** | | **YPP** | | **GPC** | |
|  | **DF** | **SS** | **MS** | **SS** | **MS** | **SS** | **MS** | **SS** | **MS** |
| **Treatment** | 1 | 0.3 | 0.35 | 22.6 | 22.6^***^ | 0.85 | 0.849^***^ | 0.1 | 0.006 |
| **Genotype** | 11 | 793.4 | 72.12^***^ | 729 | 66.27^***^ | 35.7 | 3.245^***^ | 43.76 | 3.978^***^ |
| **T x G** | 11 | 1.2 | 0.1 | 8.5 | 0.77 | 0.57 | 0.052^***^ | 0.04 | 0.003 |

**Significant Codes:** ‘^***^’ 0.001 ‘^**^’ 0.01 ‘^*^’ 0.05

DTF: number of days to flowering; DTM: number of days to maturity; SPS: number of spikelets per spike; GPS: number of grains per spike; PH: plant height; TGW: 1000-grain weight; YPP: yield per plot; GPC: grain protein content; DF: degree of freedom; SS: sum of square; MS: mean square; T: treatment, and G: genotype.

**Supplementary Table 3:** Pooled analysis of variances for different traits of 12 wheat genotypes under PN treatment

|  |  | **DTF** | | **PH** | | **SPS** | | **GPS** | |
| --- | --- | --- | --- | --- | --- | --- | --- | --- | --- |
| **S.V.** | **DF** | **SS** | **MS** | **SS** | **MS** | **SS** | **MS** | **SS** | **MS** |
| **Treatment** | 1 | 1.1 | 1.12 | 11.7 | 11.68 | 0.15 | 0.151 | 742.4 | 742.4^***^ |
| **Genotype** | 11 | 416.1 | 37.83^**^ | 1385.2 | 125.93^***^ | 37.35 | 3.4^***^ | 440.7 | 40.1^***^ |
| **T x G** | 11 | 22.1 | 2.01^*^ | 92.2 | 8.39^*^ | 15.16 | 1.38^***^ | 166.5 | 15.1^**^ |
|  |  | **DTM** | | **TGW** | | **YPP** | | **GPC** | |
|  | **DF** | **SS** | **MS** | **SS** | **MS** | **SS** | **MS** | **SS** | **MS** |
| **Treatment** |  | 19 | 19.01^***^ | 69.6 | 69.6^***^ | 0.69 | 0.6903^***^ | 0.05 | 0.049 |
| **Genotype** |  | 517.2 | 47.02^***^ | 311.89 | 28.35^***^ | 17.633 | 1.603^***^ | 117.07 | 10.643^***^ |
| **T x G** |  | 3.9 | 0.35 | 20.26 | 1.84 | 0.979 | 0.089^***^ | 0.20 | 0.019 |
|  |  | **Chl. BS** | | **Chl. AS** | | **Chl. 10DAA** | | **Chl. 20DAA** | |
|  | **DF** | **SS** | **MS** | **SS** | **MS** | **SS** | **MS** | **SS** | **MS** |
| **Treatment** | 1 | 3.58 | 3.578 | 99.52 | 99.52^***^ | 68.64 | 68.64^***^ | 0.01 | 0.014 |
| **Genotype** | 11 | 220.45 | 20.041^***^ | 250.28 | 22.75^***^ | 239.65 | 21.79^***^ | 230.98 | 20.998^***^ |
| **T x G** | 11 | 111.25 | 10.114^**^ | 39.16 | 3.56 | 20.39 | 1.85 | 3.57 | 0.325 |
|  |  | **Chl. 25DAA** | | **Chl. 30DAA** | | **N BS** | | **N AS** | |
|  | **DF** | **SS** | **MS** | **SS** | **MS** | **SS** | **MS** | **SS** | **MS** |
| **Treatment** | 1 | 10.8 | 10.81 | 0.1 | 0.06 | 4.893 | 4.893^***^ | 2.139 | 2.139^***^ |
| **Genotype** | 11 | 471.4 | 42.86^***^ | 486.1 | 44.19^***^ | 20.316 | 1.847^***^ | 9.614 | 0.874^***^ |
| **T x G** | 11 | 212 | 19.27^***^ | 0.1 | 0.01 | 2.733 | 0.248^***^ | 1.361 | 0.124^***^ |
|  |  | **N 10DAA** | | **N 20DAA** | | **N 25DAA** | | **N 30DAA** | |
|  | **DF** | **SS** | **MS** | **SS** | **MS** | **SS** | **MS** | **SS** | **MS** |
| **Treatment** | 1 | 2.153 | 2.1528^***^ | 0.002 | 0.0022 | 0.004 | 0.0044 | 0.001 | 0.0006 |
| **Genotype** | 11 | 5.442 | 0.4948^***^ | 13.154 | 1.1958^***^ | 19.753 | 1.7957^***^ | 12.34 | 1.1218^***^ |
| **T x G** | 11 | 1.223 | 0.1112* | 0.022 | 0.002 | 0.005 | 0.0004 | 0.01 | 0.0009 |
|  |  | **YP 30DAA** | | **GNC** | | **SNC** | |  | |
|  | **DF** | **SS** | **MS** | **SS** | **MS** | **SS** | **MS** |  |  |
| **Treatment** | 1 | 6942 | 6942^***^ | 0.002 | 0.0023 | 0.5101 | 0.5101^***^ |  |  |
| **Genotype** | 11 | 43840 | 3985^***^ | 3.601 | 0.3274^***^ | 1.2194 | 0.1109^***^ |  |  |
| **T x G** | 11 | 6523 | 593^*^ | 0.035 | 0.0032 | 0.2350 | 0.0214^***^ |  |  |

**Significant Codes:** ‘^***^’ 0.001 ‘^**^’ 0.01 ‘^*^’ 0.05

DTF: number of days to flowering; DTM: number of days to maturity; SPS: number of spikelets per spike; GPS: number of grains per spike; PH: plant height; TGW: 1000-grain weight; YPP: yield per plot; GPC: grain protein content; Chl. BS: chlorophyll content at booting stage; Chl. AS: chlorophyll content at anthesis stage; Chl. 10DAA: chlorophyll content at 10 days after anthesis; Chl. 20DAA: chlorophyll content at 20 days after anthesis; Chl. 25DAA: chlorophyll content at 25 days after anthesis; Chl. 30DAA: chlorophyll content at 30 days after anthesis; N BS: nitrogen content at booting stage; N AS: nitrogen content at anthesis stage; N 10DAA: nitrogen content at 10 days after anthesis; N 20DAA; nitrogen content at 20 days after anthesis; N 25DAA: nitrogen content at 25 days after anthesis; nitrogen content at 30 days after anthesis; YP 30DAA: yellow peduncle at 30 days after anthesis; GNC: grain nitrogen content; SNC: straw nitrogen content; DF: degree of freedom; SS: sum of square; MS: mean sum of squares; T: treatment, and G: genotype; SV: Source of variance.
